# Research hotspots and trends of transcranial magnetic stimulation in Parkinson’s disease: a bibliometric analysis

**DOI:** 10.3389/fnins.2023.1280180

**Published:** 2023-10-19

**Authors:** Yi-xin Wei, Liang-dan Tu, Lin He, Yi-tong Qiu, Wei Su, Li Zhang, Run-ting Ma, Qiang Gao

**Affiliations:** ^1^Department of Rehabilitation Medicine, West China Hospital, Sichuan University, Chengdu, China; ^2^Key Laboratory of Rehabilitation Medicine in Sichuan Province, West China Hospital, Sichuan University, Chengdu, China; ^3^Neurology Department, West China Hospital, Sichuan University, Chengdu, China

**Keywords:** bibliometric analysis, transcranial magnetic stimulation, Parkinson’s diseases, hotspots, research trends

## Abstract

**Background:**

Transcranial magnetic stimulation (TMS), as a non-invasive neuromodulation technique, has been widely used in the treatment of Parkinson’s disease (PD). The increasing application of TMS has promoted an increasing number of clinical studies. In this paper, a bibliometric analysis of existing studies was conducted to reveal current research hotspots and guide future research directions.

**Method:**

Relevant articles and reviews were obtained from the Science Citation Index Expanded of Web of Science Core Collection database. Data related to publications, countries, institutions, authors, journals, citations, and keywords in the studies included in the review were systematically analyzed using VOSviewer 1.6.18 and Citespace 6.2.4 software.

**Result:**

A total of 1,894 papers on the topic of TMS in PD between 1991 and 2022 were analyzed and visualized to identify research hotspots and trends in the field. The number of annual publications in this field of study has increased gradually over the past 30 years, with the number of annual publications peaking in 2022 (*n* = 150). In terms of publications and total citations, countries, institutions, and authors from North America and Western Europe were found to make significant contributions to the field. The current hotspot focuses on the effectiveness of TMS for PD in different stimulation modes or different stimulated brain regions. The keyword analysis indicates that the latest research is oriented to the mechanism study of TMS for motor symptoms in PD, and the non-motor symptoms are also receiving more attention.

**Conclusion:**

Our study offers insights into the current hotspots and emerging trends of TMS in the rehabilitation of PD. These findings may serve as a guide for future research and the application of TMS for PD.

## 1. Introduction

Parkinson’s Disease (PD) is the second most frequent neurodegenerative disorder (Thomas and Beal, 2007). Studies have been conservatively estimating that the number of people with PD will increase from 6.9 million in 2015 to 14.2 million in 2040 (Pringsheim et al., 2014; Dorsey et al., 2018). Clinical symptoms of PD are mainly resting tremor, physical dyskinesia, postural instability, gait difficulty and rigidity (Jankovic, 2008; Salmon et al., 2023; Scully et al., 2023). However, the pathogenesis of PD is still unclear, and the mainstream view is that PD is caused by a combination of genetic, environmental and aging factors that may lead to degeneration and apoptosis of dopaminergic neurons in the substantia nigra (Puschmann, 2017; Zhang et al., 2018; Marras et al., 2019). Pharmacological treatment significantly improves quality of life and functional capacity in patients with PD and L-3,4-dihydroxyphenylalanine (L-DOPA) is the most potent drug (Fahn, 2008; Jenner, 2008). However, long-term use L-DOPA leads to adverse effects including levodopa-induced dyskinesia (LID) in most patients (Cenci and Konradi, 2010; Cerasa et al., 2015).

Based on such clinical complications of PD, non-pharmacological treatments are used as attempted treatments. Transcranial Magnetic Stimulation (TMS) has received close attention as a possible treatment for PD over the past two decades (Fregni et al., 2005; Elahi et al., 2009). In 1985, Baker and colleagues conducted the first experiments of TMS in humans (Barker et al., 1985), which has been developed in the following 30 years (Hallett, 2007; Pitcher et al., 2021). TMS is defined as a magnetic stimulation technique that utilizes a pulsed magnetic field that acts on the central nervous system of the brain to alter the membrane potential of cerebral cortical nerve cells, causing them to generate induced currents that affect metabolic and neuroelectric activity in the brain, thereby inducing a series of physiological and biochemical responses (Hallett, 2000). TMS has been found to be useful as a research tool for evaluating cortical function in a variety of neuropathological states (Gilio et al., 2002; Caipa et al., 2018), and it also has a place in the treatment of a variety of neurological disorders, such as depression, chronic pain, post-stroke deficits, and PD (Shukla et al., 2016; Zhang et al., 2017; De Risio et al., 2020; Zhu et al., 2022).

As a non-invasive treatment, TMS for PD neuromodulation therapy has attracted many studies on the therapeutic management of PD. Several randomized controlled trials have explored the therapeutic effects and mechanisms of TMS for PD (Taib et al., 2019; Chung et al., 2020). Common examples of TMS on PD neuromodulation are as follows: (1) rTMS improves upper limb function in the short-term, walking performance and UPDRS III in the short-and long-terms in PD individuals and primary motor cortex (M1) is considered a more prominent stimulus point (Chung and Mak, 2016). (2) For the non-motor symptoms of PD, some studies suggest that using high frequency of rTMS on the dorsolateral prefrontal cortex (DLPFC) may be a potentially effective way to alleviate depressive symptoms (Zhang et al., 2022). (3) In addition, repetitive magnetic pulses change the excitability of the stimulation site and affect the cortical connectivity of PD individuals (Gonzalez-Garcia et al., 2011; Chung et al., 2020; Mi et al., 2020). However further studies are required to investigate optimal rTMS therapeutic protocols for PD (Chen and Chen, 2019).

Bibliometric analysis is carried out using visualization tools to analyze large volumes of published academic literature, which can be used to explore qualitatively and quantitatively the contributions of authors, countries/regions, institutions, and their partnerships. More importantly, bibliometric analysis can identify hotspots and frontiers, and predict trends in a given field, which can be an important indicator for future follow-up research. Citespace and VOSviewer are two commonly software for visualization and analysis. CiteSpace, one of the most suitable software for bibliometric analysis is developed by Prof. Chaomei Chen (Drexel University, United States) (Chen, 2004; Chen, 2006; Pan et al., 2018). Citespace is based on co-citation analysis theory and pathfinding network algorithm, which makes it easier to analyze and explore the trends and research hotspots of related disciplines (Chen and Song, 2019). VOSviewer developed by Leiden University’s Centre for Science and Technology Studies is also a software tool for visualizing bibliometric networks (You et al., 2021). In recent years, several researchers have conducted visualization analyses in the field of TMS or PD. Li et al. conducted a 20-year visualization analysis of PD acupuncture treatment, which revealed the future research potential of PD acupuncture treatment (Li X. et al., 2022). Zhang et al. conducted a 20-year bibliometric analysis of non-motor symptoms of depression and anxiety in PD, concluding that non-motor symptoms have increasingly become a hotspot for future research (Zhang et al., 2023). Liu et al. conducted a visual analysis of postural deformities in PD and summarized the current peripheral and central etiology of postural deformities in PD and rehabilitation treatment options (Zhang et al., 2023). Similarly, a study that assessed the breadth of the TMS literature base using a bibliometric approach evaluated the development over the last 30 years, helping to understand the historical progress of TMS over the last few decades (McLean, 2019).

Over the past decade, a considerable number of scholars and academic journals have focused on publishing TMS in PD. Moreover, no studies performed an innovative overview of TMS in PD neurorehabilitation through bibliometric analysis to this point. This study conducted a bibliometric analysis of TMS for PD based on records published from the inception (1900) to 2022. We used the VOSviewer 1.6.18 and Citespace 6.2.4 to identify publication patterns and emerging trends based on the Web of Science Core Collection (WoSCC) database. This article aimed to help clinicians and researchers to comprehend the issues and research hotspots related to TMS for PD and gain new insights that can guide future research and applications.

## 2. Materials and methods

### 2.1. Data collection

We use the Web of Science (WOS) database, a renowned scientific data services platform developed by Clarivate (version © 2021 Clarivate). We can retrieve the Clarivate Journal Impact Factor (IF) for the last 5 years from the WOS. Publications with related themes from the inception (1900) to 2022 were searched from the Science Citation Index Expanded (SCIE) of the WoSCC database. SCIE is a subdatabase of WoSCC, which consists of global journals of basic science research, covering neuroscience and medical research related to the theme of this study, “TMS in PD.”

The data were obtained on 15th June 2023 from SCIE. To obtain documents explicitly employing the concerning terms we performed a topical search with the query TS = (“Parkinson Disease” OR Parkinson* OR “Idiopathic Parkinson’s Disease” OR “Lewy Body Parkinson’s Disease” OR “Parkinson’s Disease, Idiopathic” OR “Parkinson’s Disease, Lewy Body” OR “Parkinson Disease, Idiopathic” OR “Idiopathic Parkinson Disease” OR “Lewy Body Parkinson Disease” OR “Primary Parkinsonism” OR “Parkinsonism, Primary” OR “Paralysis Agitans”) and TS = (“Transcranial Magnetic Stimulation” OR magnetic field therap* OR “Magnetic Stimulation, Transcranial” OR “Magnetic Stimulations, Transcranial” OR “Stimulation, Transcranial Magnetic” OR “Stimulations, Transcranial Magnetic” OR “Transcranial Magnetic Stimulations” OR “Transcranial Magnetic Stimulation, Single Pulse” OR “Transcranial Magnetic Stimulation, Paired Pulse” OR “Transcranial Magnetic Stimulation, Repetitive” OR “noninvasive brain stimulation” OR TMS OR TBS). We only selected articles or reviews in English, other document types, such as letters, commentaries, and meeting abstracts, were excluded. Finally, a total of 3,174 literature records were included.

### 2.2. Data import and deduplication

**Figure 1:**
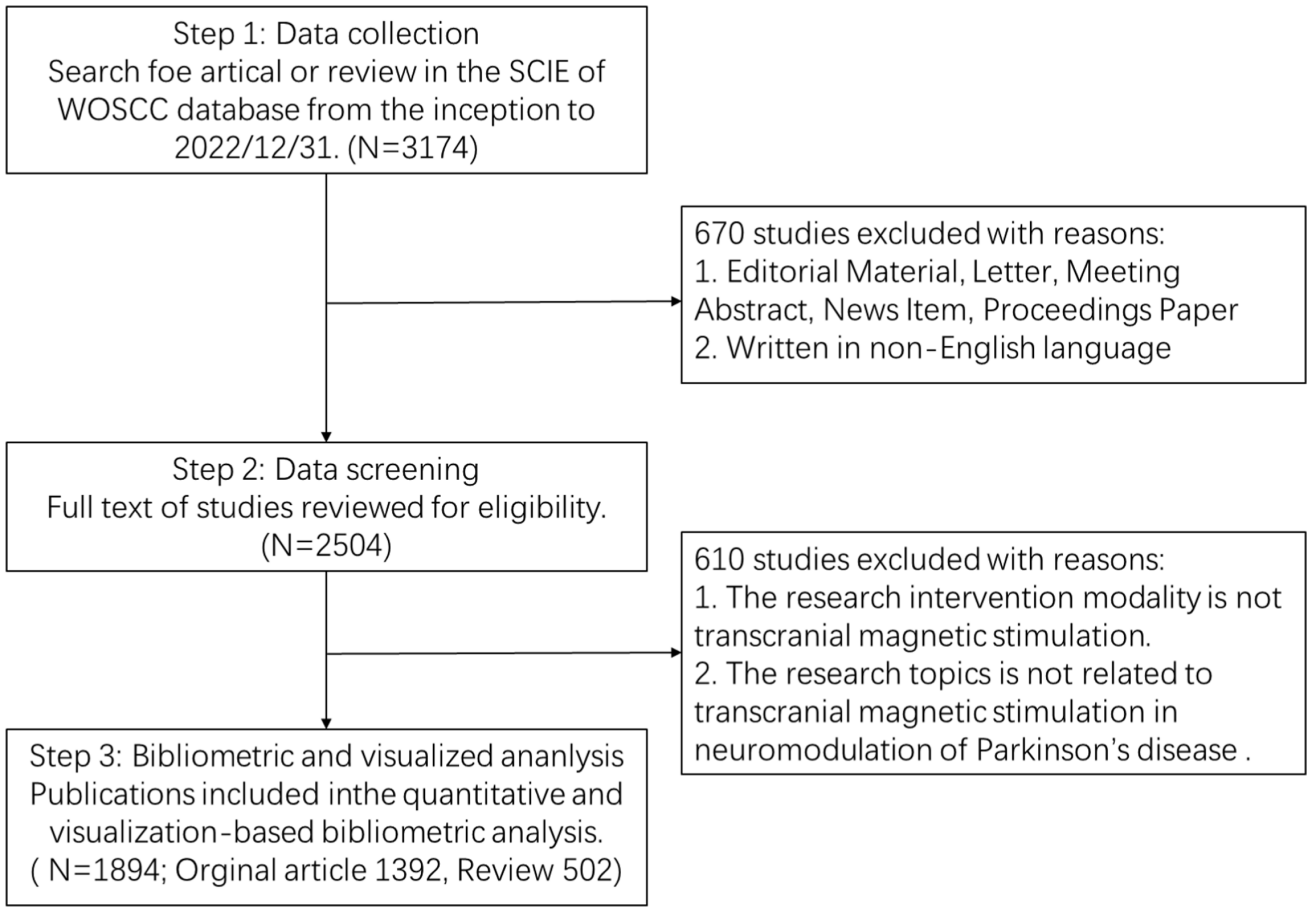
Flow chart of TMS for PD studies inclusion.

All included documents were required to undergo peer review. All bibliometric data were imported into Endnote 20 to deduplicate, and then we screened the titles, abstracts, and full texts of the included papers to identify the available studies independently based on the exclusion criteria. Exclusion criteria were as follows: (1) The intervention modality is not TMS; (2) Targeted conditions are unrelated to PD; (3) The theme of the paper is uncorrelated to the implementation of TMS in PD neuromodulation. Finally, we included a total of 1,894 articles (1,392 articles, 502 reviews) that met the requirements. The bibliometric search and analysis flowchart is presented in Figure 1.

### 2.3. Data merging

After screening and verifying through Endnote, relevant literature was handpicked from the WOS database. The plain text containing information about these documents was downloaded from the WOS database. After data download, the items of the field need to be manually merging. We identify three common scenarios that require data merging and propose solutions for each of them. These scenarios are: (1) different spellings or formats of the same country name, e.g., USA and United States of America will be merged into USA; (2) different abbreviations or variations of the same author name, which we resolve by using ORCID information and author affiliation; and (3) different terms or expressions that refer to the same concept, e.g., TMS and transcranial magnetic stimulation will be merged into transcranial magnetic stimulation.

### 2.4. Data analysis and visualization

After data deduplication and merging, the plain text will import into VOSviewer 1.6.18 and Citespace 6.2.4 for further analysis and visualization. Citespace proposes the concepts of burst detection, correlation, centrality, and heterogeneous networks, which can help to find the central point, turning point, hotspot and trend of research in related fields. VOSviewer provides text mining capabilities that can be used to build the clustering map of countries, journal, and institutions.

## 3. Results

### 3.1. Publication outputs and time trend

**Figure 2:**
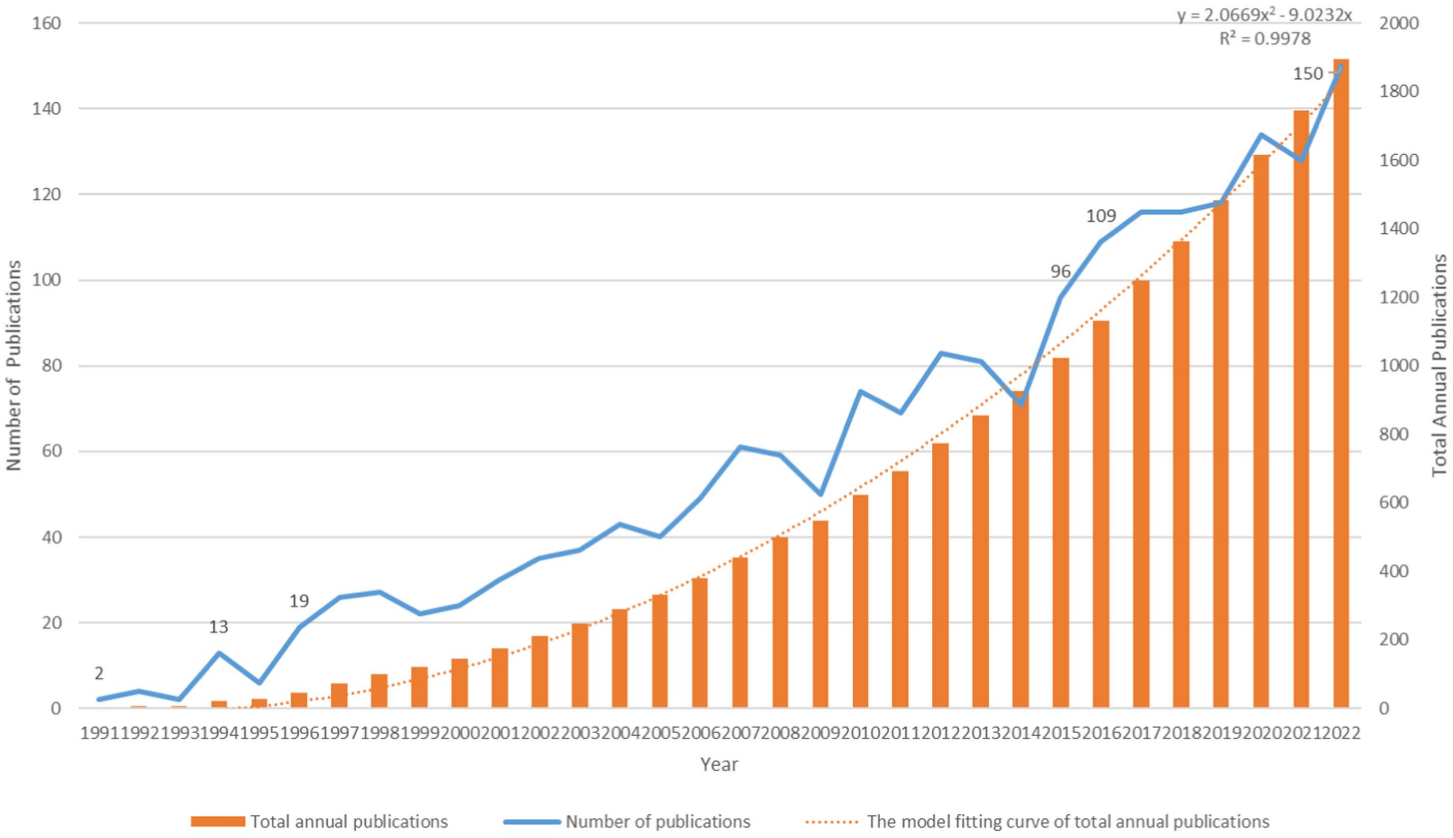
Annual publications, total annual publications, and the model fitting curve of total annual publications.

The initial search of the WoSCC database identified 31,74 publications. After excluding other literature types, limited English language, and irrelevant research topics, 1,894 papers were ultimately included in the analysis. These papers consisted of 1,392 articles and 502 reviews and were published between 1991 and 2022. The distribution and time trend of annual publication outputs and total annual publication outputs from the inception to 2022 are shown in Figure 2. The timing of publications can be divided into three phases: the infancy phase (1991–1995), the slow-growth phase (1996–2015), and the high-growth phase (2016–2022). In the infancy stage, the number of annual publications has been below 10 except in 1994. In the slow-growth stage, there is a slow but steady year-by-year upward trend from 19 papers in 1996 to 96 papers in 2015. In the high-growth stage, it maintains a high growth rate of more than 100 publications per year, and the number of annual publications increases year by year. Meanwhile, linear regression analysis showed a positive correlation (y = 2.0669 × 2 − 9.0232×, R2 = 0.9978) between the total annual number of articles. It is expected that research in this area will continue to grow in the future.

### 3.2. Distribution of countries

**Figure 3:**
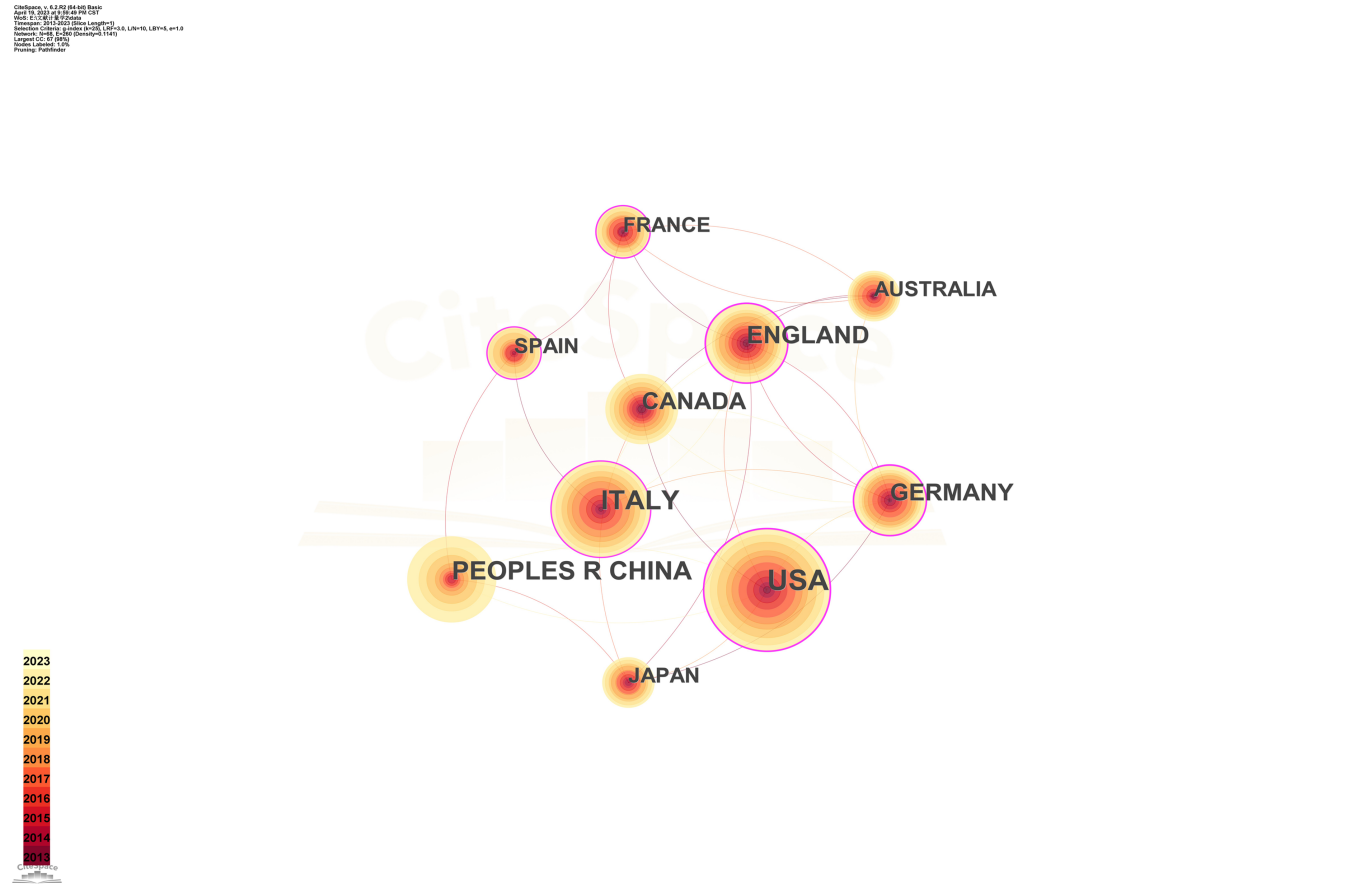
Network map of countries engaged in TMS for PD research.

The top 10 countries/regions in terms of number of publications are displayed in Table 1. Figure 3 presents a network map of countries involved in TMS research for PD. The United States has the most publications (293), followed by Italy (184), China (151), England (119), and Canada (100) have more than 100 publications. The year each country began research is represented by the different shades of the circles. The United States represents the early start of western countries in the use of TMS for PD research, and China has seen a rapid increase in the number of publications in this field in the last decade. Centrality indicates the strength of the number of connections between a node and other nodes in the entire network; high centrality means that key nodes have a strong influence on the relationships in the network. The pink outer circle in Figure 3 indicates that the centrality is greater than 0.1, indicating high centrality, and in Table 1 suggests that China (0.02) has a low central influence in this field and Germany (0.27) has the highest central influence.

**Table 1:** The top 10 countries of TMS for PD research.

Rank	Country	Count	Centrality
1	USA	293	0.22
2	Italy	184	0.13
3	China	151	0.02
4	England	119	0.21
5	Canada	100	0.08
6	Germany	99	0.27
7	Spain	57	0.15
8	Australia	56	0.09
9	Japan	56	0.01
10	France	53	0.14

### 3.3. Analysis of institutions

**Figure 4:**
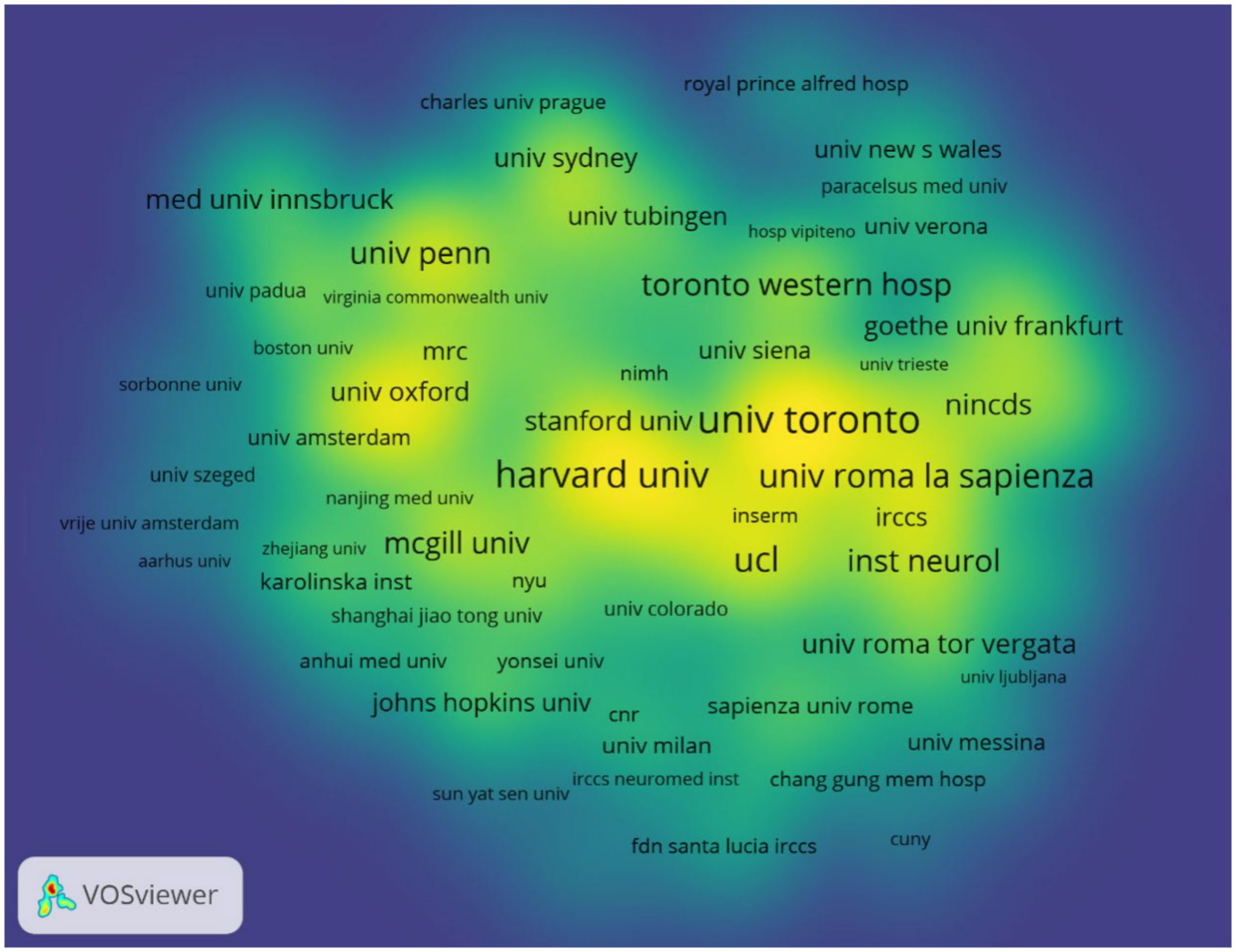
The VOSviewer network map of institutions in the field of TMS for PD.

According to author addresses, 1,888 institutions contributed to these 1,894 papers. The 10 institutions that published the most papers are listed in Table 2. The institution with the most publications is the University of Toronto (Canada, 95 papers). It was followed by Harvard University (USA, 74 papers) and the Sapienza University of Rome (Italy, 63 papers). The Sapienza University of Rome had the highest number of citations (3888) and citations per paper (61.71). Figure 4 displays the collaborative network between top institutions that apply TMS to PD neuromodulation research. The top-ranked institutions have extensive collaborative relationships with other institutions, and the yellow blocks represent the density of collaboration between institutions. We found that the collaboration between the University of Toronto, Harvard University and the Sapienza University of Rome is very close among all institutions.

**Table 2:** The top 5 productive institutions regarding the research on TMS for PD.

Rank	Institution	Country	Count	Citations	Citations per paper
1	University of Toronto	Canada	95	5,454	57.41
2	Harvard University	USA	74	5,929	80.12
3	Sapienza University of Rome	Italy	63	3,888	61.71
4	University College London	England	62	3,781	60.98
5	National Institute of Neurological Disorders and Stroke	USA	49	3,833	78.22
6	University of Tokyo	Japan	33	1,922	58.24
7	Istituto di Ricovero e Cura a Carattere Scientifico	Italy	31	1,820	58.71
8	University of Sao Paulo	Brazil	29	1,702	58.69
9	University of Sydney	Australia	28	1,507	53.82
10	University Health Network	Canada	27	705	26.11

### 3.4. Analysis of journal co-citation

**Figure 5:**
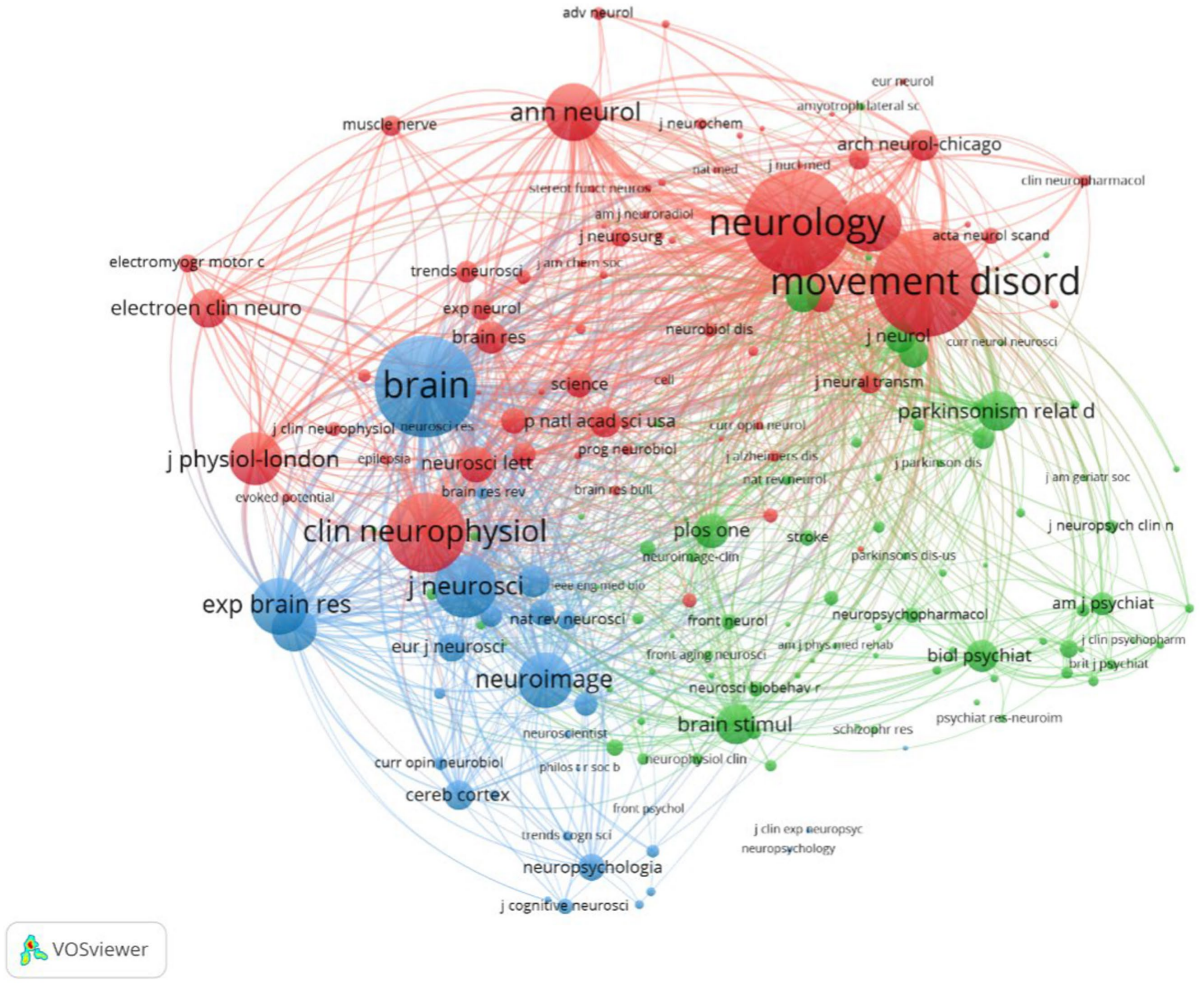
VOSviewer co-citation analysis clustering map of journal in the field of TMS for PD.

The 1,894 papers included in this analysis were published in 557 academic journals. According to Bradford’s law, core journals are those that publish papers more than one-third of all journals in the relevant field (Goffman and Morris, 1970; Weinstock, 1971; Venable et al., 2016). In this research field, there were 9 core journals, 54 related journals and 494 journals were non-related journals. Table 3 shows that the top 10 journals with the highest number of publications accounted for 37.06% (702) of all studies. Movement disorder had the highest number of publications (234), followed by Clinical neurophysiology (105), Neurology (59), and Experimental brain research (59 articles). The top 10 journals with the highest IF in the last 5 years were Brain (16.173). Five journals had an IF>5.000, four journals held an IF from 3.000 to 5.000, and one journal had an IF < 3.000. The impact of scholarly publications on a research area is determined by the number of co-citations they receive. The co-citation analysis was operated by the VOSviewer and displayed in Figure 5. The size of the nodes indicates the number of co-citations, and the lines between the nodes indicate co-citation relationships. In terms of color for the cluster analysis, the red cluster represents journals specializing in neurology, such as Movement disorder and Neurology. The green cluster represents academic journals in the field of Parkinson’s research, with Parkinsonism and Related Disorders being representative journal. Brain science neuromodulation journals, represented by Brain, are classified as blue cluster.

**Table 3:** The top 10 journals that published articles regarding the research on TMS for PD.

Rank	Journal title	Country	Count	Citations	Citations per journal	JCR	IF 5 year
1	Movement Disorders	USA	234	8,040	34.36	Q1	9.956
2	Clinical Neurophysiology	Ireland	105	5,475	52.14	Q1	4.720
3	Neurology	USA	59	4,578	77.59	Q1	11.786
4	Experimental Brain Research	Germany	59	2,454	41.59	Q4	2.193
5	Parkinsonism and Related Disorders	England	50	855	17.10	Q2	4.515
6	Frontiers in Neurology	Switzerland	47	543	11.55	Q2	3.403
7	Brain Stimulation	USA	46	1,497	32.54	Q1	9.611
8	Brain	England	35	5,371	153.46	Q1	16.173
9	Journal of the Neurologic Science	USA	35	1,089	31.11	Q2	3.580
10	Journal of Neurology	Germany	32	631	19.72	Q1	5.138

### 3.5. Analysis of authors

**Figure 6:**
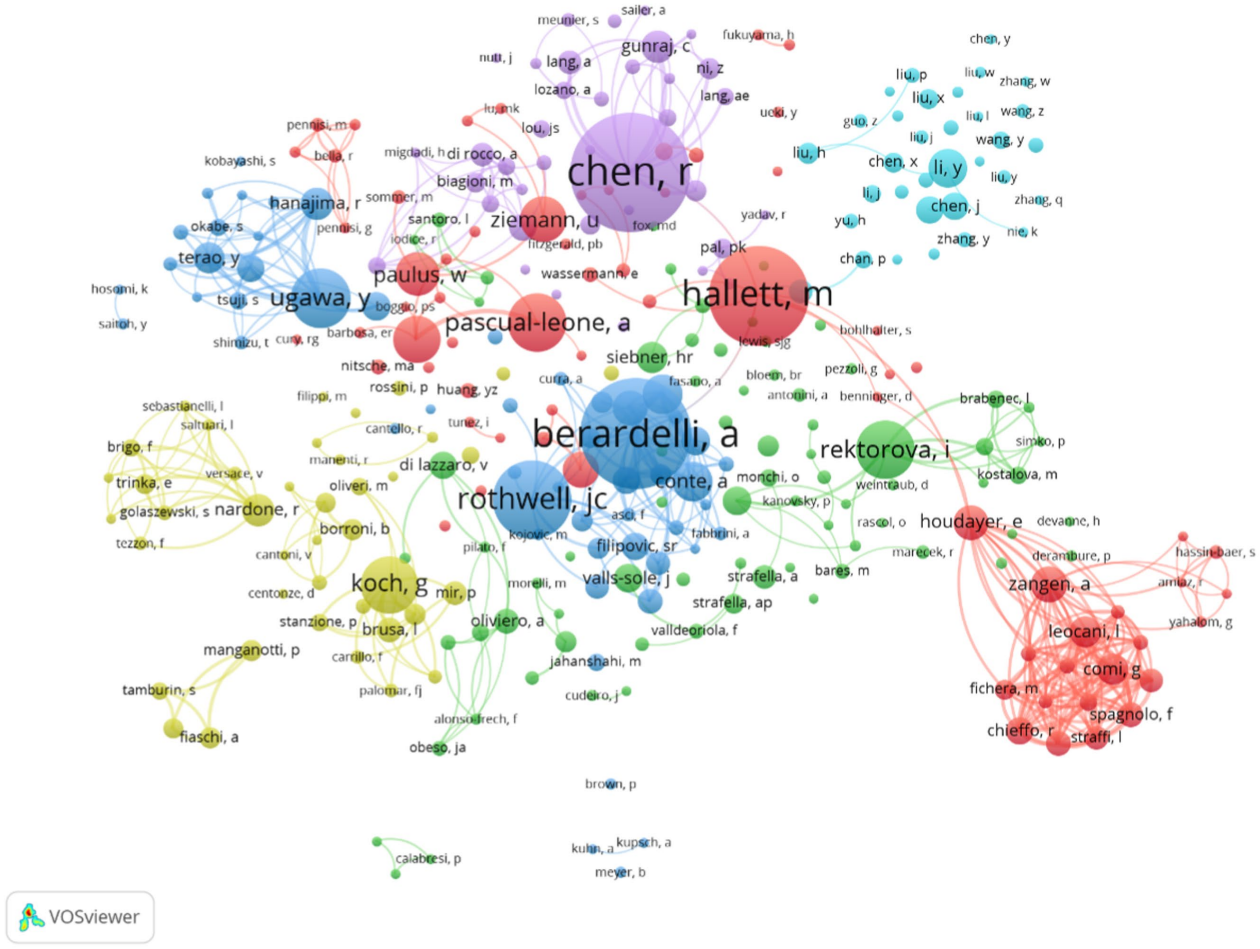
Co-author network in the field of TMS for PD.

A total of 7,659 authors were involved in the literature on neuromodulation of TMS against PD. Table 4 shows the top 10 most active authors and their relevant information. Chen Robert published 65 publications, ranking first among all authors, followed by Berardelli Alfredo (60 publications) and Hallett Mark (54 publications). The top 10 authors are scattered in different research institutions. As shown in Table 4, Hallett Mark from the National Institute of Neurological Disorders and Stroke ranked first in terms of total citations (2,243 citations). In terms of the average number of citations per paper, Pascual-leone Alvaro from Harvard Medical School ranked first. In addition, the H-index can also accurately reflect the academic achievements of authors. Rothwell John Christine is ranked first (163) in H-index and has the largest impact in the field. Figure 6 is an overlay visualization of the author co-occurrence analysis generated by VOSviewer. The graph forms major clusters centered on the top 10 authors. The collaboration between them is very close and stronger between authors in the same cluster.

**Table 4:** The top 10 authors and co-cited authors in TMS for PD research.

cRank	Author	Institution	Country	Publications	Citations	Citations per paper	H-index
1	Chen, Robert	University of Toronto	Canada	65	3,957	60.88	83
2	Berardelli, Alfredo	Sapienza University of Rome	Italy	60	4,054	67.57	21
3	Hallett, Mark	National Institute of Neurological Disorders and Stroke	USA	54	5,547	102.72	116
4	Rothwell, John Christine	University College London	England	42	1,622	38.62	159
5	Ugawa, yoshikazu	Fukushima Medical University	Japan	32	1,349	42.16	61
6	Pascual-leone, Alvaro	Harvard Medical School	USA	31	4,544	146.58	150
7	Rektorova, Irena	Masaryk University	Czech	31	578	18.65	31
8	Koch, Giacomo	Istituto di Ricovero e Cura a Carattere Scientifico	Italy	30	1,354	45.13	60
9	Fregni, Felipe	University of Sao Paulo	Brazil	25	2,496	99.84	30
10	Ziemann Ulf	University of Tubingen	Germany	24	3,374	140.58	93

### 3.6. Analysis of keywords

**Figure 7:**
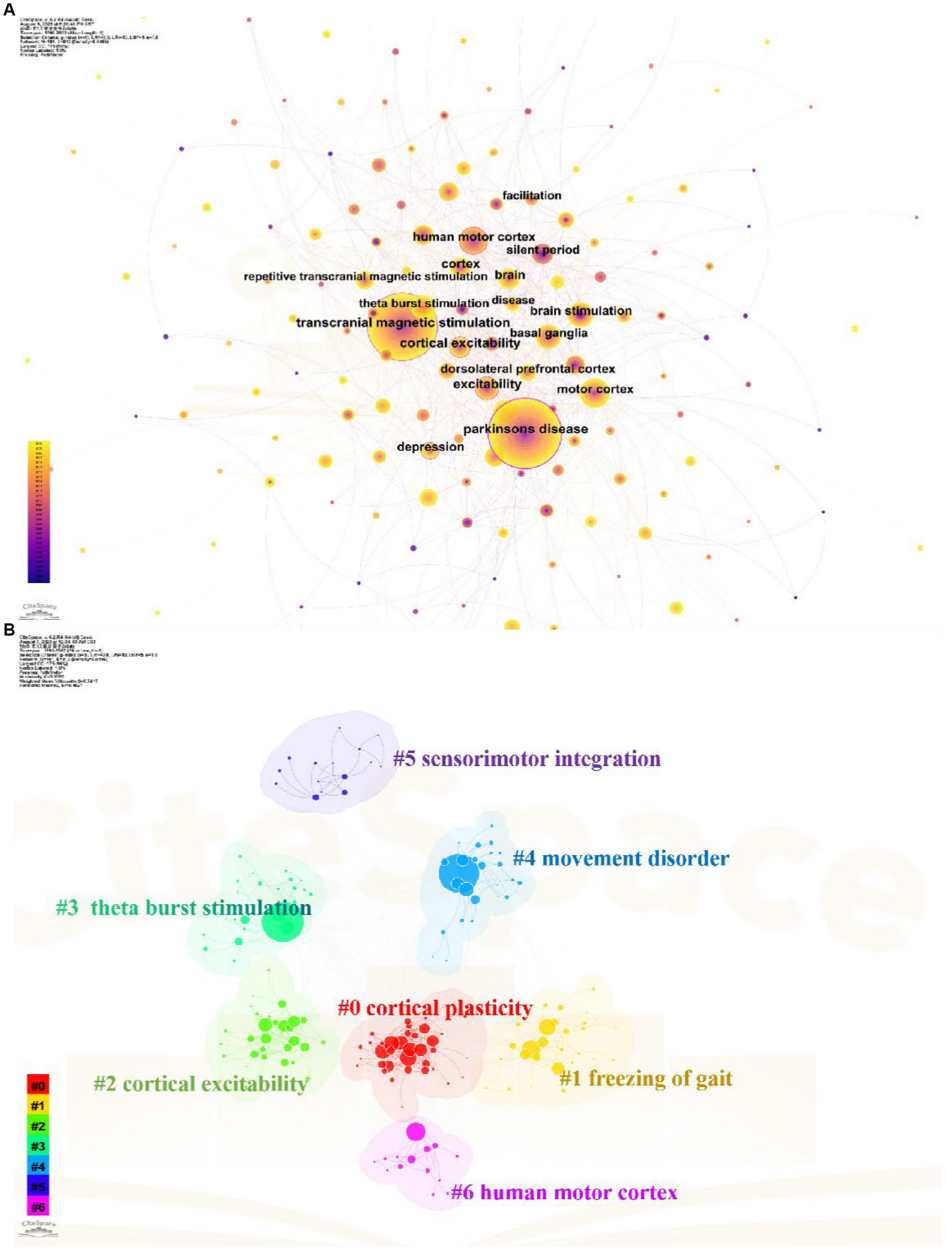
Analysis of keywords related to TMS for PD field. **(A)** The keywords co-occurrence network map. **(B)** The keywords cluster map.

Keywords are words that have substantial meaning in the center of a paper. These high-frequency or emergent keywords can reflect current topics and predict future research frontiers. As shown in Figure 7A, the top three most frequent keywords are Parkinson’s disease, transcranial magnetic stimulation and human motor cortex. Other highly frequent keywords mainly include different stimulation modes and stimulation of cortical areas in the treatment of TMS for PD. According to the different types of keywords, the keywords can be categorized into 7 clusters shown in Figure 7B. The #0 and #2 clusters mainly describe the cortical physiological changes after TMS stimulation for Parkinson’s. The #0 and #2 clusters mainly describe the cortical physiological changes after TMS over PD cortex. The #1 and #4 clusters mainly show the various directions of dysfunction research of TMS over PD. The other clusters represent the stimulation modalities of TMS, stimulation of brain regions and the treatment modes of other sensorimotor integration.

**Figure 8:**
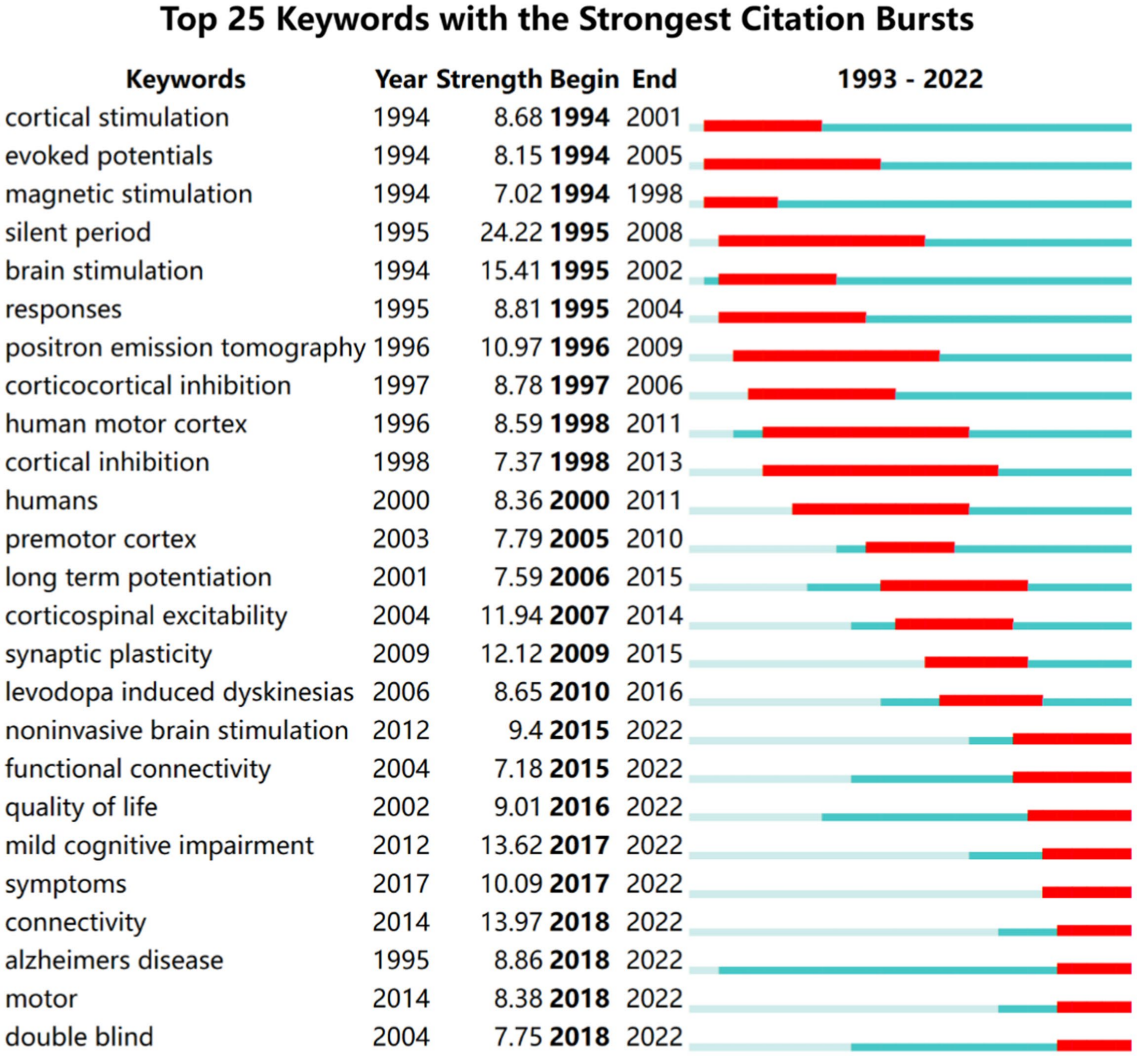
Top 25 keywords with the strongest citation bursts of the 1,894 included studies from 1993 to 2022.

Citation burst analysis is a good atlas that provides a good analysis of specific areas of research for specific year hotspots and trends (Chen et al., 2014). We used Citespace to generate the top 25 keywords with the strongest bursts and the results are shown in Figure 8. The keywords were categorized into three periods based on the time of the burst: 1993 to 2000, 2001 to 2015, and 2016 to 2022. Among these keywords silent period has the highest burst intensity. Connectivity mild cognitive impairment quality of life and motor and double blind are the most recent emergent keywords which indicates the recent research direction.

## 4. Discussion

### 4.1. General information

In the past decades, TMS has received a lot of attention and research from scholars, and the number of related studies is increasing year by year. In this bibliometric study, a total of 1,894 papers on the topic of TMS in PD were analyzed and visualized using VOSviewer and Citespace to identify research hotspots and trends in the field. The changes in research activity and productivity were evident in the publications on related topics, which can be divided into three phases. Prior to 1995, the number of papers remained relatively constant. In 1985, Baker successfully developed the first transcranial magnetic stimulator (Barker et al., 1985). However, due to the immaturity of the technology and its untapped potential for application, TMS was not widely used in PD therapy. After 1996, with the application and promotion of TMS and high-quality studies confirming its feasibility for neuromodulation, the number of papers steadily increased, attracting increasing attention from medical professionals and researchers. Since 2016, the number of annual publications has maintained a high growth rate of over 100, reaching 150 by 2022. This trend indicates that the tremendous research potential of TMS as a non-invasive and safe neuromodulation technique for the management of motor and non-motor symptoms in PD has attracted increasing attention from scholars (Dinkelbach et al., 2017; Li R. et al., 2022). So, we can anticipate that this research area will continue to be popular in the future.

Of the 10 countries with the highest number of publications in this research area, nine are developed countries and only one developing country, China, is represented. The USA, Italy, Canada, England, and Germany took the dominant places in this field. Although China is a late starter in this field of research, it has developed rapidly. Especially in the last decades, the number of publications has increased rapidly. However, the low centrality (0.02) of China indicates that there are fewer links between developing countries such as China and developed countries in this field of research and a lack of international cooperation. This is also evident from the network of institutional collaborations in Figure 4 and Table 2, which shows that the field of neuromodulation research in TMS for PD is still dominated by national institutions in Europe, the United States, and Canada, such as: the University of Toronto in Canada, Harvard University in the United States, University College London in England, and Sapienza University of Rome in Italy. Therefore, the visual analysis of countries and institutions shows that the most active and highlighted institutions were nearly from the well-established universities from developed countries with rich academic resources, so there is a certain imbalance in the exchange of academic resources between developing countries and developed countries. This phenomenon can perhaps be explained in several ways. Firstly, the developed countries started their research in the field of TMS neuromodulation earlier in 2002 or even earlier, while the developing countries, represented by China, have published more articles, and established some connections with related countries only in the last decade. Secondly, the financial constraint and inadequate attention of developing countries hinder their sustained investment in the application of TMS technologies to healthcare management. This may lead developing countries lack high-quality research results.

The distribution of author clusters is characterized by similarities with that of the country and institution clusters. The top 10 ranked authors in Table 4 are basically from research institutions in developed countries. From the network diagram of author clusters in Figure 6, it is found that Chen Robert, Berardelli Alfredo, Hallett Mark, and Rothwell John Christine constitute three clusters in the field with more extensive collaborations. Chen and Berardelli reviewed the clinical neurophysiology of movement disorders in PD, which help to advance knowledge of detecting cortical excitability with single and paired TMS (Chen R. et al., 2022). Rothwell and Berardelli assessed M1 and pre-SMA excitability in PD using TMS, which provides an innovative way to investigate motor cortical network changes of PD (Leodori et al., 2022). Mark et al. indicated 1 and 25 Hz rTMS can promotes gait training in PD by rebalancing cortical excitability (Chung et al., 2020).

The data collected from publication outputs and citations indicate that Movement Disorders is the most influential journal in this area. The journal is an official journal of the International Parkinson and Movement Disorder Society (MDS) with a 2022 impact factor of 9.698. The journal includes articles on movement disorders due to lesions of the basal ganglia, the cerebellum, and its connectivity network, including PD, chorea, and others. This has greatly contributed to our understanding of the disorders commonly seen in movement disorders and the associated brain mechanisms. For instance, the 2019 revision of evidence-based medical guidelines for managing non-motor symptoms of PD propose the short-term efficacy of TMS in ameliorating Parkinson’s depressive symptoms alongside pharmacotherapy. Nevertheless, the effectiveness of TMS still requires further evidence-based support (Seppi et al., 2019). In addition to Movement Disorders, several neuroscience journals, such as Brain, Journal of Neuroscience and Brain Stimulation, and Neurology, have also contributed high-quality research to the field. The number of papers published in the top 10 journals was more than one-third of the overall papers. Meanwhile, these active journals have a high IF, with only one journal lower than 3. Therefore, the top 10 journals of TMS for PD show high research standards with excellent clinical trials, providing reliable evidence for researchers.

### 4.2. Research hotspots and trends

The keyword is intended to help understand emerging trends and future research directions (Chen, 2006). Therefore, the following views on the future research direction hotspots of TMS for neuromodulation in patients with PD are based on the analysis of keywords.

#### 4.2.1. About TMS stimulation of cortical areas in patients with PD

An essential factor to optimize TMS treatment of PD is the chosen target site since various brain areas are implicated in PD (Eidelberg, 2009). So far, most studies have focused on the primary motor cortex (M1) or the prefrontal cortex (Fregni et al., 2005). Chou et al. suggested that high-frequency rTMS targeting M1 or low-frequency rTMS applied to other frontal regions showed significant improvement in motor symptoms in PD (Chou et al., 2015). Several studies have also proposed that stimulation of SMA can significantly improve motor symptoms such as tremor and freezing of gait in PD patients, but high-quality RCT evidence is still needed (Shirota et al., 2013; Mi et al., 2019). For the non-motor symptoms, Xie et al. (2015) point out HF-rTMS over left DLPFC in PD patients may provide possible antidepressant efficacy. However, recent studies suggest that the DLPFC may not be an ideal target site for rTMS (Chen et al., 2006). Therefore, it is essential to determine in future trials whether DLPFC is the optimal region to be involved in depression.

#### 4.2.2. About the efficacy of TMS on motor function in patients with PD

Based on the results of keyword burst and cluster analysis motor function in people with PD has received increasing attention from researchers in the past decades. This reason can be the freezing of gait is one of the common motor symptoms in patients with PD and is a major risk factor for falls, which greatly reduces mobility and quality of life (Giladi and Nieuwboer, 2008; Nutt et al., 2011; Rocha et al., 2023). Recent studies suggested that TMS has a certain improvement effect on some indicators of gait in people with PD, and this effect is not maintained in the long term, but only in the short term (Xie et al., 2020). However, Chung et al. proposed that 1 Hz and 25 Hz rTMS stimulation of the M1 could improve walking performance in people with PD in the short and long term compared to the sham rTMS group (Chung et al., 2020). Mi et al. (2018) used 10 Hz rTMS to stimulate SMA and found that the improvement in turn to sit duration lasted for at least 4 weeks, but the improvement in stride length and stride velocity was not significant. Shirota et al. (2013) also found that high-frequency rTMS had only transient beneficial effects, while low-frequency rTMS had long-term beneficial effects. Therefore, TMS has a complex impact on gait parameters in individuals with PD (Gilat et al., 2021). The patient’s equilibrium and capacity to execute intricate motor tasks are also taken into consideration, and it is strongly connected to the patient’s cognitive ability, with walking in a straight line usually necessitating less cognitive ability, while turning necessitates more cognitive capacity to modify the gait pattern (Gilat et al., 2021). Based on keyword and literature co-citation analysis it can be concluded that TMS parameters, stimulation of the cerebral cortex and maintenance effects need further investigation.

#### 4.2.3. About the mechanism of neuromodulation effect of TMS on PD

From the visual analysis of keywords in the last decade, researchers’ attention is not limited to the effect of different parameters and stimulation of different brain regions, but more and more attention is paid to the study of the mechanism of action of TMS for Parkinson’s patients.

TMS has the ability to modulate connectivity within the stimulated network and can be used to assess the excitability of motor cortex in bilateral hemispheres (Lefaucheur et al., 2004; Chen J. et al., 2022), for example, motor threshold (MT) is used to assess the excitability of cortical glutamatergic (Glu)-ergic motor neurons (Ziemann et al., 1996), motor evoked potential (MEP) size reflects the overall excitability of corticospinal pathways (Welch et al., 2020), and ipsilateral silent period (ISP) reflects the functional integrity of corpus callosum connections (Werhahn et al., 1995). Meanwhile, the combination of magnetic resonance imaging (fMRI) can also better reflect the connectivity and excitability of the brain of PD patients (Hallett et al., 2020). In combination with fMRI there is a study showing increased connectivity of the internal globus pallidus with the cerebello-thalamocortical circuit in PD tremor patients with compared to non-tremor and healthy populations (Helmich et al., 2011). Other fMRI studies have also suggested enhanced cerebellar-SMA functional connectivity in PD patients (Mueller et al., 2017). Mi et al. used rTMS over SMA in combination with fMRI to demonstrate that the alleviation of freezing of gait in PD patients was associated with normalization of brain connectivity patterns (Mi et al., 2018).

## 5. Conclusion

According to a comprehensive analysis, the use of TMS in the treatment of PD is increasing. Although North America and Europe have a significant academic influence, several institutions in developing nations, particularly China, have demonstrated limitless promise in this area. Most high-impact institutions and authors are in developed countries, indicating an imbalance in academic development. Additionally, most journals in the field have high IF, making them valuable sources of scholarly reference. There are still questions about the best modulation strategy for TMS in PD (e.g., the best choice of cerebral cortex stimulation for various motor and non-motor symptoms, the choice of stimulation intensity and stimulation duration), and the justification for the neuromodulation mechanisms used by TMS for people with PD. These clinical difficulties have not only received a lot of attention lately but will also be the subject of further study.

## Author contributions

Y-xW: Conceptualization, Data curation, Formal analysis, Methodology, Project administration, Resources, Software, Validation, Visualization, Writing – original draft, Writing – review & editing, Supervision. L-dT: Data curation, Formal analysis, Methodology, Validation, Writing – review & editing, Visualization. LH: Software, Supervision, Validation, Writing – review & editing. Y-tQ: Supervision, Validation, Visualization, Writing – review & editing. WS: Supervision, Validation, Visualization, Writing – review & editing. LZ: Supervision, Validation, Visualization, Writing – review & editing. R-tM: Supervision, Validation, Visualization, Writing – review & editing. QG: Conceptualization, Funding acquisition, Project administration, Supervision, Validation, Writing – original draft, Writing – review & editing.
